# Airport sentinel surveillance and entry quarantine for dengue infections following a fever screening program in Taiwan

**DOI:** 10.1186/1471-2334-12-182

**Published:** 2012-08-06

**Authors:** Mei-Mei Kuan, Feng-Yee Chang

**Affiliations:** 1 Chief-Secretary's Office, Taiwan Centers for Disease Control, Taipei, Taiwan, R.O.C; 2 Director-General's Office, Taiwan Centers for Disease Control, Taipei, Taiwan, R.O.C; 3 Department of Internal Medicine,, National Defense Medical Center, Taipei, Taiwan, R.O.C

**Keywords:** Imported dengue, Asymptomatic viremia, Dengue-competent hotspots

## Abstract

**Background:**

Dengue has not reached an endemic status in Taiwan; nevertheless, we have implemented a fever screening program at airports for the early detection of febrile passengers with a dengue infection. This study is intended to assess the performance of the airport screening procedures for dengue infection.

**Methods:**

We analyzed data from the national surveillance system of the Taiwan Centers for Disease Control. We included the imported dengue cases reported by sentinel airports and clinics as well as the domestic cases from 2007–2010.

**Results:**

Approximately 44.9% (95%CI: 35.73-54.13%) of the confirmed imported dengue cases with an apparent symptom (febrile) in the viremic stage were detected via the airport fever screening program, with an estimated positive predictive value of 2.36% (95% CI: 0.96- 3.75%) and a negative predictive value > 99.99%. Fluctuations in the number of the symptomatic imported dengue cases identified in the airports (X) were associated with the total number of imported dengue cases (Y) based on a regression analysis of a biweekly surveillance (i.e., n = 104, R^2^_X:Y_ = 0.61, *P* < 0.005). Additionally, the fluctuating patterns in the cumulative numbers of the imported dengue cases (X) with a 1–2 month lead time (t) was in parallel with that of the domestic dengue cases (Y) based on a consecutive 4-year surveillance (i.e., n = 48, R^2^_X(t-1):Y_ = 0.22, R^2^_X(t-2):Y_ = 0.31, *P* < 0.001) from 2007–2010.

**Conclusions:**

A moderate sensitivity of detecting dengue at the airports examined in this study indicated some limitations of the fever screening program for the prevention of importation. The screening program could assist in the rapid triage for self-quarantine of some symptomatic dengue cases that were in the viremic stage at the borders and contribute to active sentinel surveillance; however, the blocking of viral transmission to susceptible populations (neighbors or family) from all of the viremic travelers, including those with or without symptoms, is critical to prevent dengue epidemics. Therefore, the reinforcement of mosquito bite prevention and household vector control in dengue-endemic or dengue-competent hotspots during an epidemic season is essential and highly recommended.

## Background

Dengue viruses are some of the most significant arbovirus pathogens worldwide and cause endemic infections in tropical and subtropical regions. Approximately one-third of the global human population is at risk of infection [1-4]. The human incidence of dengue infection has increased dramatically over the past 50 years [1-3] due to the lack of an effective vaccine, exponential increases in international travel and shifts in global ecology. Additionally, the *Aedes aegypti* species, which is a major dengue vector, has spread considerably. Moreover, the population growth and urbanization of tropical and subtropical regions have increased the breeding sites for the mosquitoes that transmit the dengue virus [1-3]. The clinical features of dengue infection range from a mild, febrile illness (dengue fever) to fatal conditions, such as dengue hemorrhagic fever and dengue shock syndrome. The four related dengue virus serotypes have worldwide distributions, and the genotypes that are associated with increased virulence have expanded from South and Southeast Asia into the Pacific regions and the Americas [1,5,6].

Dengue is non-endemic in Taiwan, and the clusters of domestic cases have been the result of viremic travelers arriving from dengue-endemic countries [7,8]. The distinct ecologies of southern and northern Taiwan contribute to the different epidemiological patterns observed in these regions. Southern Taiwan is conducive to dengue outbreaks because of its tropical climate and the presence of *A. aegypti*, which is a major dengue vector that is accustomed to being indoors. In contrast, northern Taiwan is less threatened by the dengue virus because it has a subtropical climate and is home to *Aedes albopictus*, which is a minor dengue vector that is accustomed to being outdoors [8,9]. Furthermore, three consecutive days with temperatures below 18°C results in the abolishment of the viral transmission chain mediated by the *Aedes* mosquitoes. This climatic bottleneck effect suppresses dengue proliferation each year and significantly curbs outbreaks in southern Taiwan [8-10]. This cold climatic effect is responsible for the non-endemic status of dengue in Taiwan; however, heavy international travel in Taiwan might promote the importation of the dengue virus and viral transmission within the community.

Before 2003, the self-completion of a health card and the visual inspection of passengers were implemented at the borders of most countries to screen for infections including dengue. The emergence of Severe Acute Respiratory Syndrome (SARS) in 2003 highlighted the role of international travel in the rapid spread of infectious diseases and prompted many countries, including Taiwan, to establish border control strategies and introduce noncontact infrared thermometers (NCITs) at international airports to reduce the risk of imported infections [11,12]. However, several studies have indicated that the border control measures do not significantly contribute to the prevention of a local epidemic [12-15]. NCITs or alternative measures of border screening were found to be effective in the early detection and isolation of index cases, thus providing a short-term (7–12 day) delay in the local transmission of novel influenzas [16].

Taiwan is one of the countries that has implemented entry screening with NCITs since 2003 [11,12]. Subsequently, we expanded this noninvasive diagnostic tool to screen for various infections, including dengue, at entry borders. Using the NCITs for dengue fever screening at airports has allowed us to retrospectively analyze the datasets of detected dengue cases. In this study, we aim to assess the performance of the airport fever screening program in regard to dengue triage for self-quarantine and its potential role for active sentinel surveillance.

## Methods

### Case definitions and database analysis

Dengue is classified as a reportable infectious disease and suspected cases must be reported within 24 hours for a clinical diagnosis in Taiwan. The analyses in this study used data of the confirmed dengue cases obtained from the National Notifiable Disease Surveillance System of the Taiwan Centers for Disease Control (Taiwan CDC). The definition of a confirmed dengue case includes the positive detection of RNA, antigen or antibody by laboratory diagnoses. A domestic dengue case was a confirmed case in which the patient had not traveled in the two weeks prior to the onset of illness. An imported dengue case was a confirmed case in which the patient had traveled to dengue-endemic countries in the two weeks prior to the onset of illness. The total imported dengue cases included those inbound passengers that were diagnosed as dengue-positive by airport screening and those that were diagnosed by clinics or hospitals following entry into the community.

### Surveillance of dengue by active and passive procedures

For the comprehensive and effective surveillance of dengue infection, both active surveillance and passive surveillance were performed. The active surveillance includes fever screening at the airport, health statements from the inbound passengers, screening for contact with confirmed cases and school-based reporting. The passive surveillance refers to the hospital-based reporting system for the notification of either imported or domestic dengue cases. All relevant data and diagnostic results were reported via the web-based National Surveillance System for subsequent tracking and management.

The airport fever screening program was introduced in 2003 to detect viremic travelers who were infected with SARS, dengue or other infections [11]. In this study, we analyzed data that were collected from the International Taoyuan Airport and the International Kaohsiung Airport, which consisted of 4 or 1 entry gates in Taiwan during 2007–2010, respectively. Two thermal NCITs were set up at each entry gate, and each NCIT had an infrared-thermal camera (remote sensor device, Flir A40 or Flir P20). Travelers with an NCIT-detected temperature of higher than 37.5°C were detained at the entry gate, rechecked by quarantine officers with a symptoms survey and reassessed using an ear thermometer. Travelers with a temperature above 38°C were defined as confirmed fever cases. Subsequently, travelers who had arrived from dengue-affected areas were triaged for additional diagnostics by quarantine officers and their specimens were taken for testing with the Dengue NS1 Rapid Test Kit (Bio-Rad, USA) at the airport inspection stations. Simultaneously, duplicate specimens were sent to the central laboratory for confirmation. In the central laboratory, a rapid dengue diagnostic system based on serological [envelope and membrane–specific capture immunoglobulin M (IgM), IgG enzyme-linked immunosorbent assay] and/or nucleic acid tests (1-step real-time reverse transcription polymerase chain reaction) [8,11] were used to confirm a dengue infection.

### Statistical analysis

The analyses of the annual, monthly and biweekly infection patterns were based on the number of accumulated cases over each specified time period. The queried items (independent variables versus dependent variables) were analyzed by a regression or correlation test. To evaluate the performance of the airport fever screening in predicting the total number of imported cases, we analyzed the relationship between the number of imported cases detected by airport-screening (X) and the total number reported by airports, hospitals or clinics (Y) by a linear regression (P < 0.05 was considered statistically significant). The quantified relationship between the lead time of the imported cases (t: month) with that of the domestic cases was examined as a test trial We assumed that a unit of lead time (t) ranged from 0 to several months, and we individually tested each set of data to estimate the potential relationships. The association between the fluctuating patterns in the imported cases (Xt) and the domestic cases (Y) was measured by the Pearson’s correlation test (R _X(t):Y_; R < 0.3 was considered statistically insignificant for the correlation test) or by a linear regression analysis (P < 0.05 was considered statistically significant). Moreover, the airport fever screening program was surveyed using the positive predictive value (PPV), negative predictive value (NPV), sensitivity and specificity tests; where PPV = TP/(TP + FP); NPV = TN/(FN + TN); sensitivity = TP/(TP + FN); specificity = TN/(FP + TN); TP: true positive, the confirmed dengue importations that were detected in the airports; FP: false positive, the total of the febrile travelers who had specimens taken for diagnostics minus the confirmed dengue importations that were detected in the airports; TN: true negative, the total of all of the inbound travelers minus the febrile travelers who had specimens taken for diagnostics minus the confirmed dengue importations that were detected in the community; and FN: false negative, the confirmed dengue importations that were detected in the community. In addition to the 10 NCITs used at the airports, another 10 NCITs were used for fever screening in 9 international harbors. The influence of the entry route, e.g., by boat, was considered when the PPV and NPV were calculated by adjusting the corresponding numerators and denominators.

## Results

### Airport sentinel surveillance following fever screening for dengue infection

By implementing fever screening by NCITs at airports for the detection of febrile inbound passengers, which was confirmed by ear temperature readings, we obtained a fever prevalence that ranged from 0.08-0.10% (Table 1) among the total inbound passengers. Approximately 29.5-50.9% of the confirmed febrile inbound passengers coming from dengue-endemic areas, including Southeast Asia, South Asia, South America and Africa, were triaged by inspectors and underwent blood sampling for laboratory confirmation. Overall, 44.9% (95% CI: 35.73-54.13%) of the confirmed imported dengue cases with apparent symptoms were detected by the thermal screening program with a PPV of 2.36% (95% CI: 0.96-3.75%), an NPV of > 99.99% and a specificity of 99.97% (95%CI: 99.96-99.97%) (Table 1).

**Table 1:** The airport screening program mediated by noncontact infrared thermometers (NCITs) followed by laboratory confirmation, 2007–2010: sensitivity, specificity and predictive values

**Year**	**2007**	**2008**	**2009**	**2010**
Total inbound passengers (sample size, n)	12,508,621	12,202,392	12,499,365	14,837,391
Inbound passengers from Southeast Asia	1,164,291	471,938	1,486,357	2,729,618
Confirmed febrile passengers^1^	11,118	12,158	12,286	12,553
Fever prevalence among total passengers %	0.09	0.10	0.10	0.08
Specimens taken for diagnostics^2^	5,654	4,946	3,706	3,956
Dengue importations detected in the airport fever screening program ^3^	72	100	108	126
Dengue importations detected in harbor screenings^4^	0	1	2	3
Dengue importations reported from communities	107	125	95	175
Sensitivity of the airport fever screening program %	40.22	44.44	53.20	41.86
Specificity of the airport fever screening program %	99.96	99.96	99.97	99.97
PPV^5^ of the airport fever screening program %	1.28	2.03	2.90	3.22
NPV^6^ of the airport fever screening program %	>99.99	>99.99	>99.99	>99.99

1. Confirmed febrile passengers were detected by NCITs (37.5**°C**) followed by ear thermometers (more than 38**°C**) in 2 airports.

2. Confirmed febrile inbound passengers from dengue-endemic countries, mainly Southeast Asia and South Asia.

3. The cumulative results of the International Taoyuan Airport and the International Kaohsiung Airport.

4. Dengue importations reported from fever screening in 9 harbors with 10 NCITs were not enrolled as true positives in calculating the PPV and NPV of airport fever screening.

5. PPV: positive predictive value of the airport fever screening program.

6. NPV: negative predictive value of the airport fever screening program.

**Figure 1:**
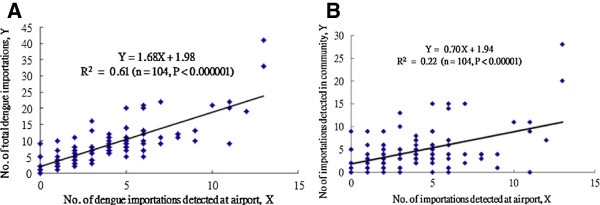
**Epidemiological trends of the dengue importation into Taiwan.** (**A**) The scatter plots show the relationship between the number of imported dengue cases, according to the viremic status as measured by a biweekly surveillance of the airport screening program, and the number of total dengue importations into Taiwan from 2007–2010. The straight line represents a line fitted by the means of a least squares regression. The coefficient of determination, R^2^, was estimated to be 0.61 (n = 104, *P* < 0.0001). The 95% confidence interval of the slope is 1.42-1.94. (**B**) The scatter plots show the relationship, as measured by a biweekly surveillance, between the number of dengue importations detected in the airport screening program and the number of dengue importations detected in the community from 2007–2010. The straight line represents a fitted line by the means of a least squares regression. The coefficient of determination, R^2^, was estimated to be 0.22 (n = 104, *P* < 0.0001). The 95% confidence interval of the slope is 0.44-0.96.

From 2007–2010, a majority of the confirmed dengue-infected travelers were arriving from dengue-endemic regions in South or Southeast Asia, such as Indonesia (21.0-35.1%), Vietnam (20.1-32.0%), Thailand (5.0-13.0%), the Philippines (9.0-12.3%), Cambodia (4.1-8.0%), Malaysia (2.0-4.1%), Singapore (1.1-3.4%), India (0–1.1%) and South America (0–0.7%). Moreover, the cumulative numbers of the imported dengue cases that were detected at the airports (X) were found to be significantly associated with the total number of imported dengue cases (Y) using a linear regression analysis based on a biweekly surveillance from 2007–2011 (Y = 1.68X + 1.98 with a 95% CI of 1.42-1.95, R^2^ = 0.61, n = 104, *P* < 0.0001; Y: biweekly total of the number of imported cases of dengue; X: biweekly number of the imported dengue cases that were screened at the airports) (Figure 1A). In an analysis of the respective year surveillance data, the biweekly association (n = 26) was demonstrated by an R^2^ value that ranged from 0.35 in 2007 to 0.72 in 2010 (Y = 1.21X + 3.53 with a 95% CI of 1.92-0.51, R^2^ = 0.35, *P* = 0.002 in 2007; Y = 1.60X + 2.49 with a 95% CI of 2.24-0.95, R^2^ = 0.52, *P* <0.0001 in 2008; Y = 1.21X + 2.38 with a 95% CI of 0.93-1.50, R^2^ = 0.75, *P* < 0.0001 in 2009; Y = 2.08X + 1.33 with a 95% CI of 1.54-2.62, R^2^ = 0.72, *P* <0.0001 in 2010).

### The non-endemic characteristics of dengue

**Figure 2:**
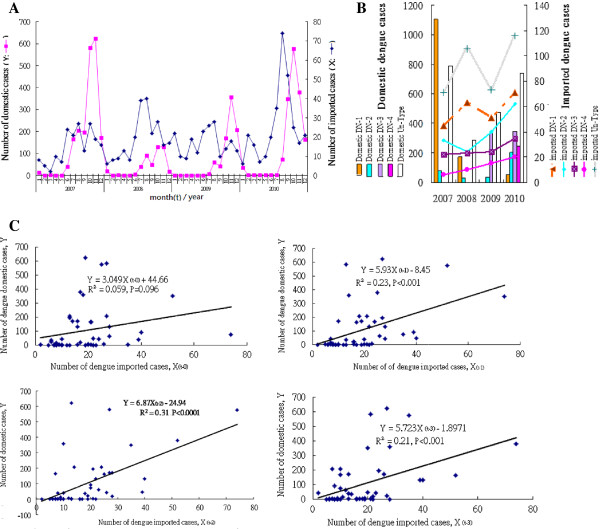
**Trends of the dengue epidemics and the relationships among the cases in Taiwan from 2007–2010.** (**A**) A comparison of the fluctuations of the monthly number of imported (Xt) vs. domestic cases (Y). (**B**) The diversity in the annual dominant dengue serotypes among the domestic and imported cases. (**C**) The scatter plots show the relationship of the number of imported dengue cases (Xt-0), (Xt-1), (Xt-2) or (Xt-3) vs. the number of domestic dengue cases, as detected by a monthly surveillance (Y). The respective straight lines represent the lines fitted by the means of the least squares regressions. The coefficients of determination, R^2^, were estimated to be 0.059, 0.21, 0.31, or 0.21 (n = 48) with respective 95% confidence intervals for the slopes being −0.57-6.67, 2.68-9.19, 3.81-9.93, or 2.46-8.98. (**C**) The diversity in the annual dominant dengue serotypes among the domestic and imported cases.

The non-endemic status of dengue in Taiwan can be confirmed by examining the epidemiological patterns from 2007–2010. First, the outbreak of domestic dengue cases was temporally aggregated with a slight increase in June, a peak from September to November, a and a gradual decrease in December; the lowest level was reached from January to February. Furthermore, there were several months (February to May) in which no domestic cases were observed in 2007–2010 (Figure 2A). Second, there were irregularities in the annual dominant dengue serotypes among the imported and domestic cases (Figure 2C), and diversity in the outbreak magnitude and location were observed (Table 2). Third, the monthly fluctuation patterns in the number of cumulative imported dengue cases (X) with a 1–3 month lead time (t: month) were associated with those of the domestic dengue cases (Y), based on a consecutive 4-year surveillance according to the Pearson’s rank test (n = 48, R_X(t-0):Y_ = 0.24, R_X(t-1):Y_ = 0.47, R_X(t-2):Y_ = 0.56, R_X(t-3):Y_ = 0.45 and R_X(t-4):Y_ = 0.22) (Figure 2A). In addition, according to a regression test with a lead time of 1–3 months, the monthly cumulative number of imported dengue cases (X) had a pattern in parallel with the domestic dengue cases (Y), based on a consecutive 4-year surveillance (n = 48; R^2^_X(t-0):Y_ = 0.04, *P* = 0.097; R^2^_X(t-1):Y_ = 0.22, *P* = 0.0008; R^2^_X(t-2):Y_ = 0.31, *P* = 0.00008; R^2^_X(t-3):Y_ = 0.20, *P* = 0.002; R^2^_X(t-4):Y_ = 0.05, *P* = 0.156) (Figure 2B).

**Table 2:** Geographical distribution of the number of imported dengue cases identified by active and passive surveillance and the number of domestic dengue cases in Taiwan, 2007–2010

		**Imported dengue cases**								**Domestic dengue cases**			
		**Active surveillance**^**1**^				**Passive surveillance**^**2**^							
		**Dengue positive**				**Dengue positive**							
^3^ Region	**City/ County**	2007	2008	2009	2010	2007	2008	2009	2010	2007	2008	2009	2010
Northern	Taipei City	12	18	17	22	17	28	13	36	0	20	0	2
Northern	Taipei County	14	22	12	23	13	27	4	36	0	12	1	15
Northern	Yilan County	1	1	0	2	1	0	18	1	0	0	0	0
Northern	Keelung City	1	2	2	1	2	2	2	2	0	1	0	0
Northern	Kinmen County	0	0	1	0	0	0	11	1	0	0	0	0
Northern	Lienchiang County	0	0	0	0	0	0	4	0	0	0	0	0
Northern	Taoyuan County	7	18	23	23	5	11	4	15	0	2	1	1
Northern	Hsinchu County	2	4	3	3	0	5	6	5	0	0	0	1
Northern	Hsinchu City	1	2	2	0	0	0	0	2	0	0	0	0
Northern	Miaoli County	2	1	1	4	1	3	7	3	0	0	0	0
Central	Taichung County	5	6	6	16	10	12	2	19	0	1	0	0
Central	Taichung City	0	1	7		5	3	8		2	0	0	0
Central	Nantou County	2	1	3	3	2	2	0	2	0	0	10	0
Central	Changhua County	2	3	6	6	2	3	0	3	0	0	0	0
Central	Yunlin County	0	3	2	1	3	1	1	4	2	0	0	0
Southern	Chiayi County	1	0	2	4	7	0	0	4	0	0	0	1
Southern	Chiayi City	0	2	0	1	0	0	1	3	0	0	0	0
Southern	Tainan County *	3	5	3	9	9	4	2	9	1459	23	8	380
Southern	Tainan City *	10	4	2		11	2	0		345	4	2	107
Southern	Kaohsiung County*	4	1	4	8	5	3	2	23	40	98	125	83
Southern	Kaohsiung City *	5	3	9		7	12	0		141	326	623	984
Southern	Pingtung County*	0	3	4	1	6	6	5	6	1	1	76	10
Southern	Penghu County	0	0	0	0	0	0	0	0	0	0	0	0
Eastern	Hualien County	0	1	1	1	1	1	1	1	0	0	0	0
Eastern	Taitung County	0	0	0	1	0	0	4	0	0	0	1	0
	Total	72	101	110	129	107	125	95	175	1,990	488	847	1,584

1. Active surveillance: imported dengue cases detected in border screening.

2. Passive surveillance: imported dengue cases detected in the community.

3. *Dengue hotspot; Merging of the city and county jurisdictions in Taichung, Tainan and Kaohsiung since 2010.

### The impact of the imported dengue cases on domestic epidemics in dengue-competent hotspots

We analyzed a total of 5,823 dengue cases in Taiwan from 2007–2010, with 15.7% of those being imported cases (Table 2). The domestic dengue outbreaks were clustered mainly in the metropolitan areas of Tainan and Kaohsiung. These areas were the most compatible for dengue endemics because of their tropical climate and the presence of *A. aegypti*, and they accounted for 99.4% of the domestic cases reported in southern Taiwan from 2007–2010 (Table 2). We detected a geographical heterogeneity of dengue outbreaks within Taiwan. In southern Taiwan, there was a positive association between the annual accumulated numbers of imported and domestic cases from 2007–2010, i.e., the annual cumulative number of domestic dengue cases = 45.5 times the annual cumulative number of imported dengue cases minus 4.58 (n = 20, R^2^ = 0.53, *P* < 0.05) (Table 2). These numbers were based on the annual surveillance of five local dengue hotspots (Kaohsiung City/County, Tainan City/County and Pintung County). In contrast, only sporadic domestic dengue cases were observed in the urbanized areas of northern Taiwan, where *A. albopictus* is the only established mosquito species and a subtropical climate is present. In the metropolitan areas of northern Taiwan, the relationship between the annual numbers of imported (X) and domestic (Y) dengue cases was low, with less statistical significance (e.g., n = 8, Y = 0.32 X - 6.27, R^2^ = 0.39, *P* = 0.099, in the Taipei area). However, from 2007–2010, the percentage of the annual cumulative number of dengue cases that were importations was 37.99% in North Taiwan (Taipei, Yilan and Keelung), higher than 24.21% in southern Taiwan (Chiayi, Tainan, Kaohsiung and Pintung) (Table 2).

The annual cumulative numbers of dengue importations reported from clinics (passive surveillance) or detected at the airports (active surveillance) were associated with the numbers of domestic cases in 2007–2010 among the five hotspots (Table 2) in southern Taiwan. An analysis of the dataset according to the geographical areas (25 counties/cities) indicated that there were significant correlations between the annual cumulative number of dengue importations identified at the airports (X) and the number of dengue importations reported from community clinics (Y) (n = 96, Y = 0.93X + 1.208, R^2^ = 0.57, *P* < 0.0001) (Table 2).

## Discussion

### The role of airport screening in dengue sentinel surveillance and entry quarantine

By implementing the airport fever screening program followed by laboratory confirmation, nearly half of the imported symptomatic dengue cases were detected at entry, i.e., 40.2% (72/179) in 2007, 44.3% (100/226) in 2008, 52.9% (108/204) in 2009 and 41.5% (126/304) in 2010 (Tables 1 and 2). The active detection of dengue importations in airports offers the opportunity to target symptomatic dengue cases for early self-quarantine at the entry border, which could potentially help to prevent some of the local transmission chains. The routine measurement of body temperatures using infrared thermal monitors at international airports enables mass-screening for febrile passengers who can be further subjected to triage. The passengers who arrive from areas of dengue outbreak and who are determined to be infected with the dengue virus are required to perform health self-management for two weeks, which includes self-quarantine and limitations on community activities. These hygienic measures are intended to block transmission of the virus and prevent the large-scale spread of infections within the community.

In this study, we find that airport dengue screening also offers an opportunity to create a model for predicting the potential magnitude of all dengue importations (R^2^_X:Y_ = 0.61, n = 104, *P* < 0.0001, 95% CI: 1.42-1.94), based on a consecutive 4-year biweekly dengue surveillance (Figure 1A). However, the coefficients of determination of the biweekly dengue surveillance (R^2^_X:Y_) varied among the respective years from 0.35 in 2007 to 0.72 in 2010; this result suggests that a prediction-fulfilment should be depended on well-controlled daily practices. Moreover, in terms of the regression relations of dengue importations detected in the airport vs. those detected in the community, the R^2^ = 0.57 was higher in the yearly surveillance (based on data derived from Table 2) than that of the biweekly surveillance with an overlapping 95% CI for the slopes (Figure 2C), indicating that a confounding of the putative reporting timing might have occurred.

Moreover, the peak time period of the domestic outbreaks, with 0 to several months (t) of lag following dengue importation, might signal an alert for the domestic outbreaks in Taiwan; however, there are some other factors that should be taken into consideration. First, the R^2^_X(t-n):Y_ values of the regression analysis were low based on a consecutive 4-year surveillance (n = 48; R^2^_X(t-n):Y_ = 0.059-0.31) and the respective slopes varied greatly, i.e., changes in the rate of Y (domestic cases) were dependent on changes in X (dengue importations) with a 95% CI that ranged from −0.57 to 9.93 (Figure 2C). Second, the incubation time of dengue is approximately two weeks [17]; however, variations in the lag of the peak timing in local dengue epidemics in this study may have been influenced by multiple factors, including the seasonal and ecological activity of the *Aedes* vectors. The above findings imply that rather than the imported cases triggering the domestic dengue outbreaks, other factors might play critical roles in affecting the magnitude of the epidemics, such as the distribution, proliferation and activity of mosquitoes which are influenced by ecological factors [9,10]; meteorological factors [18]; hygiene education for mosquito density control; and human protection from mosquito bites [19]. These findings highlight the importance for critical measures in dengue control in regard to preventing mosquito bites and reducing mosquito activity.

Furthermore, a distinct impact of the dengue importations on community epidemics was observed due to ecological heterogeneity. In northern Taiwan, the number of annual imported dengue cases was slightly higher than in southern Taiwan (Table 2). However, the impact of the annual dengue importations on domestic dengue cases was less significant (n = 8, R^2^ = 0.39, *P* = 0.09), even in the metropolitan area of urban Taipei. The presence of *A. albopictus*, a minor dengue vector accustomed to being outdoors, in this area resulted in a limited transmission of the virus. In contrast, in southern Taiwan, a significant impact was observed in the 5 hotspots (n = 20, R^2^ = 0.53, *P* < 0.05). Therefore, the viremic cases of asymptomatic, mild, latent or secondary dengue infection can simultaneously or subsequently become new infectious sources with the potential to induce severe epidemics in southern Taiwan by *A. aegypti*, a major dengue vector accustomed to being indoors.

### Limitations of airport screening for local epidemics

Different pathogens have different characteristics, the infectious peak for SARS cases is observed after the onset of symptoms, whereas the infection associated with the influenza virus begins a few hours before the onset of symptoms [12-15] and the viremia of dengue begins one day before the onset of febrile symptoms [1,20]. Thus, entry screening is considered more effective at blocking the transmission of SARS than other agents. Moreover, a major and common challenge of utilizing fever screening at borders to reduce the risk of introducing various infectious agents into a country is the bypassing of the temperature threshold by afebrile patients who import viral particles with latent (not yet symptomatic), mild and asymptomatic infections [12-15]. The current screening methods for symptomatic infections at entry borders through real-time RT-PCR and/or serum tests for dengue diagnostics are limited by a poor NCIT threshold. Thus, latent, mild or asymptomatic dengue cases that bypass the NCITs represent a source of new dengue outbreaks each year. The improvement of procedures that are aimed at identifying and isolating these cases or those that promote the education about mosquito bite prevention by using repellents are thus essential for dengue prevention in dengue-competent regions.

Another limitation of this study is the lack of a negative control to compare the impact of border intervention based on self-reported fever, symptoms inspected or the fever screening program via NCIT on mitigating community dengue epidemics. Studies have reported a higher sensitivity of entry screening for influenza using NCITs than self-reporting at points of entry into the healthcare system [21] and on the potential of using NCIT for mass influenza screening [22]. Another study reported that entry screening measures such as thermal scanners, health declarations or medical checks could delay the local transmission of the pandemic influenza A for approximately 7–12 days [16]. However, we previously observed that there was no significant impact on the magnitude of dengue epidemics in the community between conducting entry inspections during pre-airport (by self-reported questionnaires) and post-airport fever screening [9]. These findings suggested that while border measures are established to mitigate the triggering of a dengue outbreak, integrated measures for reducing the transmission of mild, latent and asymptomatic viremic carriers of dengue and preventing the spread of secondary domestic infections should simultaneously be enforced.

The other potential limitations of this retrospective study are listed below. First, other than the airport entry routes (the International Taoyuan Airport and the International Kaohsiung Airport), approximately 0–0.9% of the dengue importations detected at the borders (i.e., 0, 1, 2 and 3 cases) were introduced by boats via the 9 ports screened by NCITs from 2007–2010 (Table 1). However, we had excluded these boat-mediated dengue importations in the calculations of the PPV for the airport fever screening. Second, other than the standard detection of cases by the NCIT setting at temperature 37.5°C, the few cases that might have been screened under a conditional alarm temperature at 36.6°C or with alternative screening methods, such as symptom-inspecting or self-reporting, were not completely excluded in our calculation and might cause a bias. Nevertheless, this influence was relatively small. Third, the significant associations between the annual and monthly fluctuations of the dengue importations and the domestic cases by the regression test suggest that utilizing the cumulative data (*i.e.*, mix of all of the serotypes) might be misinterpreted at an individual level.

### The implications for airport screening as an epidemic control policy and the importance of reinforcing public hygiene education

After July of 2003, the world was declared free of SARS; however, fever screening trials in airports were expanded in Taiwan and later implemented as part of an active surveillance program for multiple infectious pathogens, such as dengue, chikungunya, malaria, influenza and enteric bacteria [11]. In addition to the detection of dengue fever, which is one of the most frequently imported reportable diseases every year in Taiwan, this active surveillance policy provided other sentinel functions. For example, there were 13 imported chikungunya infections [23] detected among febrile travelers by this active surveillance from 2006–2009; therefore, these systems conferred an increasing awareness of the threat of imported pathogens.

Our evaluation of the routine border screening for dengue using NCITs yielded a low PPV, which suggested a low cost-effectiveness and raised questions about the effectiveness of the available screening measures to detect a well-known disease at international borders. Despite the presence of devices with improved performance and affordable implementation costs, there is still a possibility for infected travelers to not be detected by screening and subsequently trigger an outbreak, which would cause a loss of public confidence in the pandemic response [12-15]. Rather than investing in active screening by NCITs, which requires a large number of specimens to be collected to detect rare infectious cases at airports [14], several studies have suggested that focusing more on public hygiene education and the prevention of mosquito bites would be beneficial. In terms of reducing costs, we suggest that alternative supporting measures should be taken, such as developing tools for rapid diagnostics with better sensitivity to replace the commercial kits. Another step should be replacing NCITs with self-reporting measures to reduce the number of specimens taken during the non-endemic periods and non-epidemic seasons while vector activity is low.

The proportion of dengue cases that are symptomatic ranges from 10-50%, meaning that approximately 50-90% of all dengue infections are asymptomatic, only a fraction of which are reported [3,24]. The frequency of measures taken by tourists to protect against mosquito bites is less than 45% [23]. Among travelers, approximately 25-57% of the dengue importations are accompanied with symptoms [25]; therefore, 43-75% of the truly asymptomatic or mild symptomatic dengue cases may become sources for additional dengue infections. In this study, approximately 45% of the symptomatic dengue importations could be detected by airport quarantine; however, the other symptomatic dengue importations were latent (not yet symptomatic infections) and were only discovered after the symptoms appeared or the patients sought medical attention. Many of the symptomatic dengue importations were not reported [3]. Dengue viremia begins 1 day before the onset of symptoms and lasts for 7–12 days after the onset of symptoms [1,17,20,26]. In Taiwan, the outbreak patterns of confirmed domestic dengue cases are usually temporally aggregated and correlated with the seasonal changes in the populations of *A. aegypti*. Because dengue is transmitted through bites from infected female *Aedes* mosquitoes, especially the primarily indoor vector *A. aegypti* that needs blood to mature their eggs, a community outbreak may occur. Therefore, we suggest the following interventions: 1) travelers to Southeast Asia, South America, and Africa should perform vector control behaviors to prevent vector borne diseases; 2) tourists should take steps to control vector spread for at least two weeks after returning from epidemic areas to stop the spread of the virus to their neighbors and family because they may be asymptomatic carriers; and 3) citizens living in areas of southern Taiwan where *A. aegypti* mosquitoes exist, such as Tainan and Kaohsiung, should perform vector control interventions to reduce virus transmission and prevent people who have been infected by a certain serotype of dengue virus from being reinfected by a different serotype and progressing to dengue hemorrhagic fever. These precautions should be taken especially during the daytime and evening hours when the activity of *Aedes* mosquitoes peaks [19].

## Conclusions

A moderate sensitivity of 44.93% and a PPV that ranged from 1.28-3.22% were obtained for airport fever screening in this study. Our findings indicated some limitations of the airport fever screening programs for preventing the introduction of dengue. However, these procedures might help to target some symptomatic dengue importations for an immediate self-quarantine that might mitigate some local dengue transmissions. In addition, a border intervention could provide an active sentinel surveillance to provide an alert for the potential magnitude of the dengue importations. As a secondary function to the alerting role, airport screening might ideally delay the viral transmission time and mitigate the domestic spread of dengue before the cold climatic effect can abolish the activity of the *Aedes* mosquito. However, the disproportionate annual cumulative number of imported vs. domestic dengue infections in dengue hotspots suggests that the viremic carriers of asymptomatic and secondary infections could become new threatening sources for the induction of severe epidemics the summer through the autumn seasons (the epidemic seasons) by the principal vector *A. aegypti*. Therefore, to curb potential dengue transmission, we strongly recommend reinforcing mosquito bite prevention among travelers or residents who are returning from dengue-endemic areas, or those who are living in dengue-competent or dengue-endemic hotspots.

## Competing interests

The authors declare that there were no conflicts of interest or funding and ethical issues involved.

## Authors' contributions

MMK and FYC designed the research. MMK conducted data collection and data analysis as well as drafted the manuscript. MMK and FYC revised the manuscript. Both authors read and approved the final manuscript.

## Pre-publication history

The pre-publication history for this paper can be accessed here:

http://www.biomedcentral.com/1471-2334/12/182/prepub
